# Nomophobia and Psychological distress among the Saudi Population

**DOI:** 10.1371/journal.pdig.0000779

**Published:** 2025-03-26

**Authors:** Noara Alhusseini, Jamil Alghanem, Salsabil Haque, Samanta Mohammed Shahin, Mohammad Alsaeed, Wael Kalou, Adel Kalou, Sara Alnasser, Majed Ramadan, Khadijah Ateq

**Affiliations:** 1 College of Medicine, Alfaisal University, Riyadh, Saudi Arabia,; 2 King Abdullah International Medical Research Centre (KAIMRC), King Saud bin Abdulaziz University for Health Science (KSAU HS), Riyadh, Saudi Arabia; Polytechnic Institute of Porto: Instituto Politecnico do Porto, PORTUGAL

## Abstract

**Introduction:**

Smartphones have become a defining feature of the 21st century, fundamentally transforming the way we live and interact. However, the pervasive use and growing dependence on these devices have led to increasing concerns about their impact on mental health. The rise of smartphone addiction, often manifesting as anxiety, irritability, and a feeling of melancholy, has contributed to the rapid increase in nomophobia, a term describing the fear of being without a mobile device. This phenomenon is increasingly linked to psychological distress as our reliance on smartphones continues to deepen.

**Aim:**

This study seeks to determine the prevalence of nomophobia and psychological distress symptoms and their relationship among the adult population of Saudi Arabia.

**Method:**

A cross-sectional survey was done among the adult population of Saudi Arabia, including Saudis and non-Saudis. An online validated survey was distributed via social media channels. SAS 9.4. was used for data analysis. Frequencies and percentages were used to display the prevalence, and the chi-square test was used for associations. A p-value <0.05 was used to determine significance.

**Result:**

A total of 704 Saudi and non-Saudi adults completed the questionnaire. The mean nomophobia score among all participants indicated a moderate level at 73.71, while the mean psychological distress score reflected a mild disorder at 22.08. Saudis reported a statistically higher significant mean nomophobia score than non-Saudis (p-value <0.0001). Participants residing in the Eastern region were significantly more prone to nomophobia (p-value 0.0003), and to psychological distress (p-value 0.004).

**Conclusion:**

The study reveals that men and Saudi nationals are particularly affected by nomophobia, likely due to their higher reliance on smartphones. Saudi nationality, educational attainment, and residing in the Eastern region of Saudi Arabia are considered predictors for nomophobia and psychological distress.

## Summary

Smartphones have revolutionized communication and daily life, but their excessive use has led to growing concerns about digital dependence and mental health. Nomophobia, the fear of being without a mobile device, is an emerging public health issue linked to psychological distress. In this study, we assess the prevalence of nomophobia and its association with psychological distress among adults in Saudi Arabia. Using a cross-sectional survey of 704 participants, we found that moderate levels of nomophobia were common, with significant associations observed based on nationality, gender, education, and geographic region. Saudi nationals, males, and individuals residing in the Eastern region exhibited higher nomophobia scores. Notably, our findings demonstrate a strong correlation between nomophobia and psychological distress, reinforcing concerns about the psychological burden of excessive smartphone reliance. Given the high global prevalence of smartphone dependency, our study emphasises the need for targeted digital health interventions to mitigate the psychological impacts of mobile technology use.

## Introduction

Smartphones have become indispensable in modern life, with many people relying on them for everyday tasks [1]. Remarkably, children and teenagers spend an average of 7.5 hours per day on screens [2]. Saudi Arabia ranks third globally in smartphone usage [3]. The smartphone has revolutionized the 21st century, offering unparalleled convenience and a vast array of features, making it a device that can seemingly do it all [4]. Its versatility enables people worldwide to communicate swiftly at any time. Beyond texting, emailing, and calling, users can book appointments, order food, plan vacations, and study using these devices [4]. However, despite the numerous benefits, excessive smartphone use can negatively impact well-being [4]. Smartphone addiction and dependence have become prevalent issues in today’s society [4]. People rely on smartphones for communication, entertainment, and information, which can lead to a dependence on these devices [4]. This dependence can cause individuals to experience withdrawal symptoms when separated from their phones, such as anxiety, irritability, restlessness, and in more extreme cases, depression and nomophobia [4,5].

The fourth edition of the Diagnostic and Statistical Manual of Mental Disorders (DSM-IV) describes nomophobia as a condition tied to modern digital life [6]. It is characterized by feelings of unease, anxiety, or stress when someone is unable to use their phone or computer [6]. Simply put, it is the fear of being without a mobile phone or being unable to use it, is a relatively new term that refers to the anxiety associated with the lack of smartphone accessibility [5,7]. Symptoms of nomophobia can include sweating, shaking, and heart palpitations [8]. Some common behaviors of nomophobia include utilizing several devices, checking their phones regularly for notifications, and sleeping with their phones close by [8]. A study shows that 71% of people usually sleep near their mobile phones [8]. In addition, a study found that individuals with nomophobia use multiple devices and chargers, and are constantly on high alert for notifications [9]. Sixty-six percent of the world’s population suffers from nomophobia [10]. Moreover, studies in Saudi Arabia and Jordan suggest that about (51.2%) of respondents were diagnosed with nomophobia [11].

Psychological distress defines the overwhelming emotions experienced by an individual in response to stressors [12]. It reflects stress, anxiety, and depression [12]. These emotions are experienced daily due to any kind of acute stress and would have a long-lasting impact on one’s mental health [12]. A cross-sectional study carried out in Jeddah, Saudi Arabia reported that there was a significant difference in the distribution of subjects with different levels of anxiety across gender (p<0.05) [13]. There was a high prevalence of depression, anxiety, and stress associated with gender, nomophobia levels, and residence type [13].

The intersection of nomophobia and psychological distress is a significant area of concern for public health [14]. The pervasive nature of smartphone dependence highlights its potential to exacerbate mental health issues, particularly in vulnerable populations such as adolescents and young adults [15]. Excessive smartphone use and associated nomophobia can disrupt sleep patterns, impair social relationships, and reduce productivity, all of which contribute to psychological distress [14,16]. Moreover, the high prevalence of nomophobia worldwide underscores the urgency of addressing this issue, especially as it correlates strongly with increased anxiety, depression, and stress [17,18]. In regions like Saudi Arabia, where smartphone usage is among the highest globally, understanding and mitigating the effects of nomophobia becomes even more critical to prevent widespread mental health repercussions [8,19]. Previous research has largely concentrated on the prevalence and contributing factors of nomophobia and psychological distress; however, this study is the first to explore the relationship between the two. This highlights the critical importance of the issue, as the social, economic, and health-related consequences of untreated psychological distress linked to smartphone overuse emphasize the need for targeted interventions and policy measures.

## Methods

This is a cross-sectional study conducted among the adult population of Saudi Arabia, including Saudis and non-Saudis. An online survey was distributed using social media channels, including WhatsApp, LinkedIn, Twitter, and Facebook. A snowball sampling method was used. Initially, a small group of eligible participants were approached and invited to participate in the study. Upon consenting to participate, these initial participants were requested to identify or refer other individuals within their networks who also met the study’s inclusion criteria. A snowball effect, allows the recruitment of additional participants through the referrals of prior participants. To minimize potential biases associated with snowball sampling, efforts were made to diversify the initial seed participants to capture a broader range of perspectives and reduce the overrepresentation of specific subgroups. Additionally, the recruitment process continued until the data were deemed as representative as possible. It was implemented with careful ethical considerations to ensure voluntary participation and respect for privacy. Participants were informed that referring others was entirely optional, and they were instructed to avoid sharing personal information about potential referrals without consent.

The sample size was measured using the estimated annual population of Saudi Arabia, which is approximately 37 million [12], and using a 95% confidence level, and 5% margin of error. The calculated sample size was 385. The questionnaire was completed by 704 participants from January 22, 2024, to March 14, 2024. Participation was voluntary, and written consent was obtained from all participants. The survey consists of 3 sections. Section 1 includes demographic data such as gender, age, marital status, region of residency, and employment status. Section 2 includes the nomophobia questionnaire (NMP- Q), considering, NMP-Q Score = 20 nomophobia is absent, 21 ≤ NMP-Q Score < 60 is mild level, 60 ≤ NMP-Q Score < 100 is moderate, and 100 ≤ NMP-Q Score ≤ 140 is severe Level. Section 3 includes a questionnaire for psychological distress (Kessler’s scale) in both English and Arabic. The cut-off scores that are used as a guide for screening for psychological distress, are 0 - 19 Likely to be well, 20 - 24 likely to have a mild disorder, 25-29 likely to have a moderate disorder, and 30 - 50 likely to have a severe disorder. Regarding the validity of the survey, both surveys are previously validated and available for the public’s use. The NMP-Q has proven to be a valid and reliable instrument for evaluating the severity of nomophobia [20,21]. The findings of a study evaluating the Kessler scale showed that is a reliable and valid tool for assessing psychological distress [22].

The preliminary descriptive table included a range of variables, such as age, gender, marital status, educational background, employment status, nationality, monthly income, region of residence, nomophobia scores, and psychological distress scores. For the subsequent multivariate analyses, certain adjustments were made to enhance the feasibility of regression analyses. Specifically, we amalgamated age categories, combining 50-59 and 60 and older into a single category. Additionally, the north and south regions were consolidated into an ‘other regions’ category. In the case of marital status, we simplified it to a binary classification, distinguishing between married and unmarried individuals. For the purposes of model construction, our primary dependent variables were the nomophobia score and the psychological distress score. All other demographic variables were treated as independent variables in our analyses.

To examine the reliability or internal consistency of the survey’s answers, a Cronbach’s alpha test was employed and resulted in a reliable alpha coefficient (0.87). In the initial univariate analysis, we conducted descriptive statistics to explore the relationship between nomophobia and psychological distress scores concerning both age and gender. A t-test was applied for binary independent variables, while an ANOVA test was employed for independent variables with more than two categories. To ensure the validity of these tests, we examined the assumptions of normality and homogeneity of variance using the Shapiro-Wilk test, Levene’s Test For homogeneity, and visualization through box plots [23]. For the subsequent multivariate analysis, a generalized linear model was employed to investigate the associations between nomophobia and psychological distress scores and various demographic factors. Data management and statistical analyses were performed using SAS 9.4. The significance threshold was set at α = 0.05.

### Ethical consideration

Ethical approval for the study was granted by the Institutional Review Board (IRB) at King Abdullah International Medical Research Center (IRB approval number: IRB/2921/23). The study adhered to the principles outlined in the Declaration of Helsinki and other relevant ethical guidelines, ensuring the highest standards of research ethics and integrity.

Participation in the study was completely voluntary, and participants were informed about the study’s objectives, methodology, and their rights. This included the right to withdraw from the study at any time without any consequences or the need to explain. To avoid any undue influence, participants were made aware that no financial or material compensation was provided for their participation.

To safeguard participant privacy, no identifying data was collected, ensuring anonymity. All responses were anonymized and stored securely. Access to the data was restricted to the authorized research team, trained in handling sensitive information in compliance with institutional and national data protection regulations.

The data collected was used strictly for research purposes, with all findings reported in aggregate to prevent the identification of individual participants. By maintaining transparency and ethical rigor, the study upheld the trust and welfare of its participants while contributing to scientific understanding.

## Results

**Fig 1:**
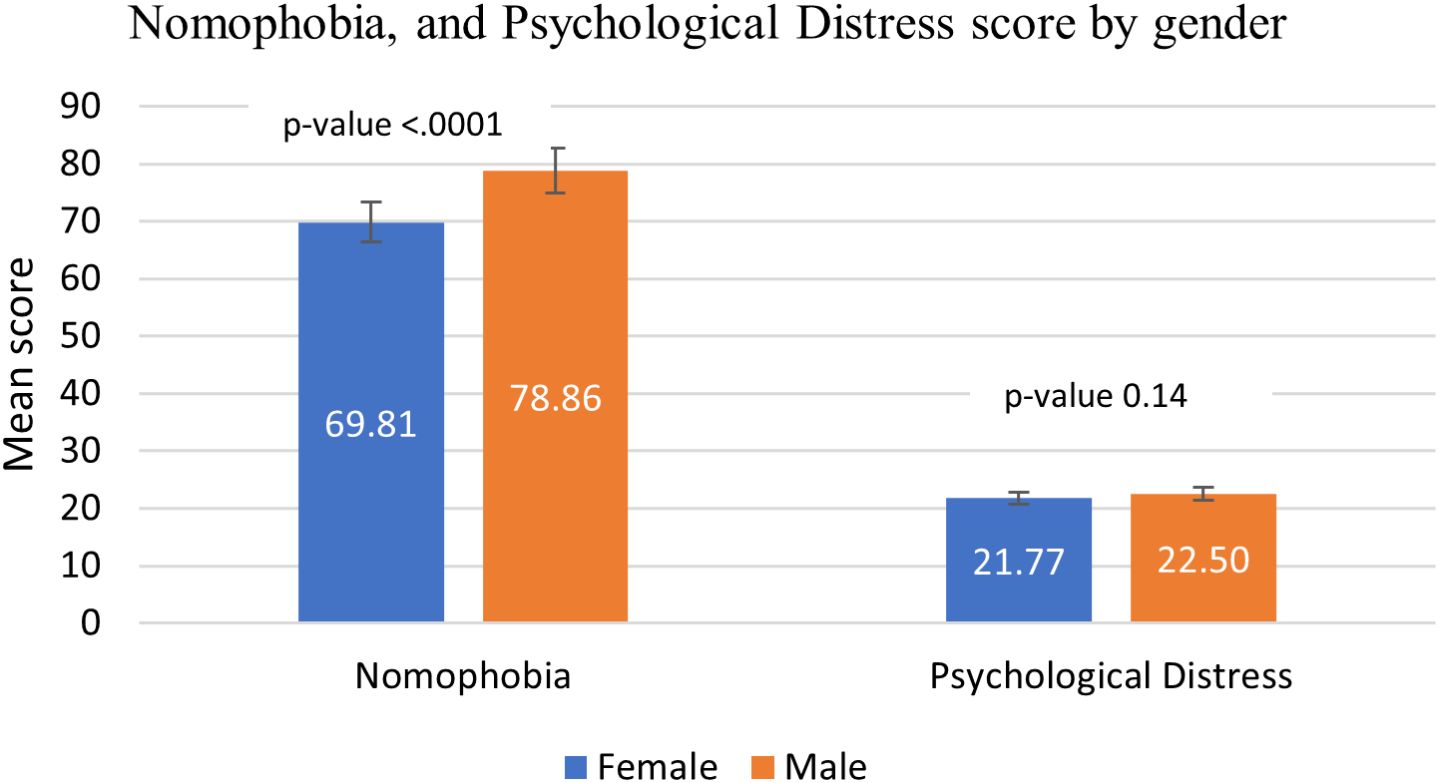
Mean scores of nomophobia and psychological distress by gender. Males had significantly higher nomophobia scores than females (p < 0.0001), while psychological distress scores showed no significant difference between genders (p = 0.14). Error bars represent standard error.

**Fig 2:**
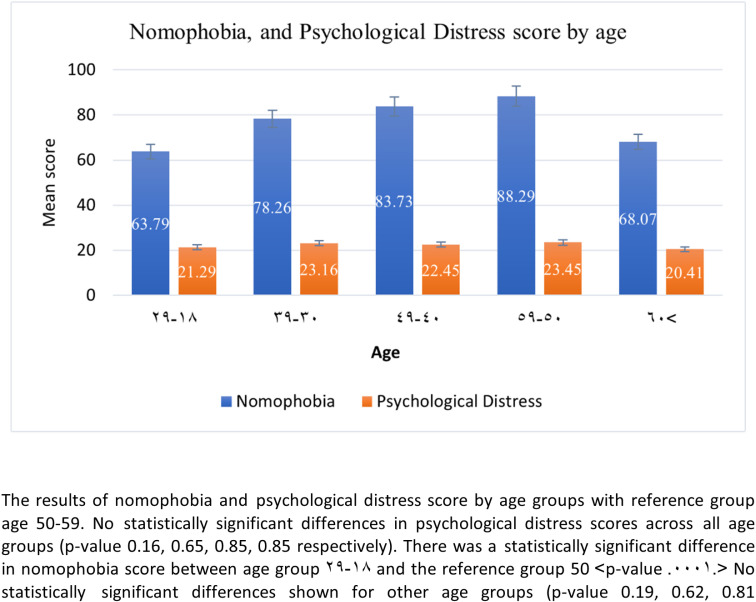
The results of nomophobia and psychological distress score by age groups with reference group age 50-59. No statistically significant differences in psychological distress scores across all age groups (p-value 0.16, 0.65, 0.85, 0.85 respectively). There was a statistically significant difference in nomophobia score between age group 29-18 and the reference group 50 (p-value < 0.001). No statistically significant differences shown for other age groups (p-value 0.19, 0.62, 0.81).

A total of 704 participants completed the surveys. Nearly half of the participants fell within the 18-29 age group, were single, and resided in the Eastern region (45%, 48.29%, and 46.02%, respectively). More than half of the participants were Saudi nationals, females, and held high school or lower degrees (66.67%, 58.43%, and 57.1%, respectively). Participants had a moderate nomophobia score of **73.71** and a mild psychological distress score of **22.08** (Table 1). The univariate analysis of nomophobia and psychological distress scores by gender and age group revealed a statistically significant difference in nomophobia scores between males and females (Fig 1). However, there was no significant difference in psychological distress scores across all age groups (p-values 0.16, 0.85, 0.65, and 0.86 for respective age groups) (Fig 2). A statistically significant difference in nomophobia scores was observed between the 18-29 age group and the reference group (50 years and older), with the reference group reporting the highest score of **88.29** (p < 0.0001) (Fig 2).

**Table 1:** Characteristics of the participants.

	Sample size (%) ^1^
**Total**	N= 704
**Age**	
**18-29**	320 (45.39)
**30-39**	152 (21.56)
**40-49**	121 (17.16)
**50-59**	81 (11.48)
**>60**	29 (4.11)
**Missing**	1 (0.14)
**Gender**	
**Male**	289 (41.52)
**Female**	407 (58.43)
**Missing**	9 (0.05)
**Marital status**	
**Single**	340 (48.29)
**Married**	339 (48.15)
**Divorced**	11 (1.56)
**Widowed**	10 (1.42)
**Missing**	4 (0.56)
**Employment status**	
**Employed**	261 (37.07)
**Student**	260 (36.93)
**Retired**	124 (17.61)
**Unemployed**	57 (7.7)
**Missing**	2 (0.28)
**What is your nationality?**	
**Saudi**	470 (66.76)
**Non- Saudi**	232 (32.95)
**Missing**	2 (0.28)
**What is your monthly income?**	
**9,999 Saudi Riyal or less**	281(39.91)
**10,000-19,999 Saudi Riyal**	146 (8.27)
**More than 20,000 Saudi Riyal**	120 (17.04)
**I prefer not to answer**	102 (14.48)
**I don’t have monthly income**	50 (7.1)
**Missing**	5 (0.71)
**What is the level of your education?**	
**High School Or Less**	402 (57.1)
**Undergraduate Degree**	224 (31.81)
**Graduate Degree**	73 (10.36)
**Missing**	5 (0.71)
**Region**	
**Eastern Region**	324 (46.02)
**Central Region**	305 (43.32)
**Western Region**	50 (7.1)
**North Region**	10 (1.42)
**South Region**	9 (1.27)
**Missing**	6 (0.85)
**Nomophobia Score**	
**Mean, Median, Standard Deviation**	73.71, 73, 28.44
**Psychological Distress Score**	
**Mean, Median, Standard Deviation**	22.08, 20, 8.69

^1^N (%) Sample Size and Percentage

The multivariate analysis was conducted to examine the association between nomophobia scores, psychological distress scores, and demographic factors. Two significant predictors of psychological distress were identified: educational status and region of residence. Psychological distress scores varied significantly across educational attainment levels, with participants holding graduate degrees reporting lower scores compared to those with a high school education or less (mean difference: -5.01; p = 0.0003) (Table 2). Similarly, participants residing in the Eastern region had a statistically significant higher mean psychological distress score compared to other regional groups (mean difference estimate 2.58; p-value 0.004) (Table 2).

**Table 2:** Predictors of Psychological Distress, And Nomophobia Scores.

	Psychological Distress Score			Nomophobia Score		
	Estimate	Standard error	P-value^1^	Estimate	Standard error	P-value^1^
**Age**						
**18-29**	-0.84	1.23	0.49	-2.61	3.08	0.39
**30-39**	Reference	Reference	Reference	Reference	Reference	Reference
**40-49**	-1.28	1.1	0.24	0.37	2.75	0.89
**50>**	-0.74	1.18	0.53	-0.05	2.96	0.98
**Gender**						
**Male**	Reference	Reference	Reference	Reference	Reference	Reference
**Female**	-0.74	0.69	0.28	-10.07	1.72	<.0001
**Marital Status**						
**Not Married**	Reference	Reference	Reference	Reference	Reference	Reference
**Married**	-0.44	1.09	0.68	-0.67	2.71	0.8
**Employment Status**						
**Employed**	0.81	1.25	0.52	-2.61	3.22	0.41
**Student**	1.51	1.41	0.58	4.51	3.71	0.22
**Retired**	-0.96	1.79	0.28	4.53	4.6	0.31
**Unemployed**	Reference	Reference	Reference	Reference	Reference	Reference
**What Is Your Nationality?**						
**Saudi**	0.59	0.92	0.52	13.46	2.34	<.0001
**Non- Saudi**	Reference	Reference	Reference	Reference	Reference	Reference
**What Is Your Monthly Income?**						
**9,999 Saudi Riyal Or** **Less**	0.39	1.15	0.71	-1.64	2.96	0.57
**10,000-19,999 Saudi** **Riyal**	0.17	1.18	0.88	-1.96	3.02	0.51
**More Than 20,000** **Saudi Riyal**	1.71	2.21	0.83	-1.07	3.11	0.72
**I Prefer Not To** **Answer**	Reference	Reference	Reference	Reference	Reference	Reference
**I Don’t Have Monthly Income**	-0.29	1.59	0.53	-4.48	4.09	0.27
**What Is the Level of Your Education?**						
**High School or Less**	Reference	Reference	Reference	Reference	Reference	Reference
**Undergraduate** **Degree**	-0.66	0.81	0.41	-1.35	2.04	0.51
**Graduate Degree**	-5.01	1.38	0.0003	-10.95	3.47	0.001
**Region**						
**Eastern**	2.58	0.91	0.004	27.12	2.31	<.0001
**Central**	-1.35	1.36	0.32	-4.83	3.4	0.15
**Other Regions** ^2^	Reference	Reference	Reference	Reference	Reference	Reference

^1^ **generlized Linear Regression Model.**

^2^ **Other Regions (Central, North, And South)**

**Fig 3:**
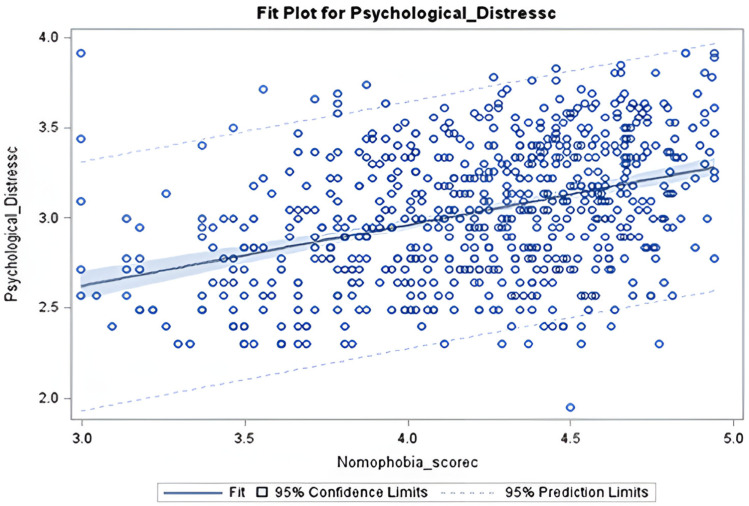
Scatter plot with a fitted regression line showing the relationship between Nomophobia Score (x-axis) and Psychological Distress Score (y-axis). A positive correlation suggests that higher nomophobia scores are associated with increased psychological distress.

For nomophobia scores, four significant predictors were identified: gender, nationality, educational status, and region. Females had a statistically significant lower mean nomophobia score compared to males (mean difference estimate -10.07; p-value <0.0001). Participants with graduate degrees reported lower nomophobia scores compared to those with a high school education or less (mean difference: -10.95; p = 0.001) (Table 2). Saudi nationals had a statistically significant higher mean nomophobia score compared to non-Saudi participants (mean difference estimate 14.46; p-value <0.0001). Participants from the Eastern region had a significantly higher mean nomophobia score compared to those from other regions (mean difference: 27.12; p < 0.0001) (Table 2). The parameter estimate for the Nomophobia score is **0.11008**, meaning that for each one-unit increase in the Nomophobia score, the expected value of the outcome variable increases by **0.11008** units. Overall, the parameter estimates indicate a statistically significant positive relationship between the Nomophobia score and the psychological distress score, with higher Nomophobia scores linked to higher psychological distress scores (p < 0.0001). (Table 3), (Fig 3).

**Table 3:** Relationship of Nomophobia and Psychological Stress.

Parameter	Estimate	Standard Error	t Value	Pr> |t|
**Intercept**	1.601	0.13	12.08	<.0001
**Nomophobia’s Score**	0.33	0.031	10.84	<.0001

**Linear Regression Model, P <.0001**

## Discussion

Numerous studies have investigated various aspects of the increasing cell phone usage among the population and have discovered significant effects on mental and physical health. The present research aimed to study the prevalence of nomophobia in Saudi Arabia and its effect on psychological distress. The findings support our hypothesis, which aligns with social addiction theory. The theory provides a framework for understanding how certain behaviors, such as excessive use of mobile phones, could lead to addictions that negatively impact social and psychological well-being. Within this context, nomophobia can be examined as a form of social addiction, where individuals become excessively reliant on their mobile devices for social interactions, validation, and emotional support. This reliance can lead to psychological distress. Overall, it can be deduced that there is a positive relationship between nomophobia and psychological distress among Saudis.

The results of this study show a moderate level of nomophobia among participants. Consistent with this, an observational study in multiple countries found that about 70% of the study population had moderate to severe Nomophobia, while about 20% had severe Nomophobia [9]. This study has indicated a higher prevalence of nomophobia in males compared to females. This is interesting because most of the other scientific evidence is contradictory. Cross-sectional research conducted at the University of Jeddah (N=335) found that females have a significantly higher incidence of nomophobia than males [24]. Many other research studies have stated similar findings [25–27].

Surprisingly, the result of this study indicated a higher mean nomophobia score among the 50-year-old and older group than the 18–29 age group. In contrast, a systematic review of 108 English and Spanish studies found that younger participants are generally more prone to nomophobia [18]. Moreover, the remark is supported by a quantitative study conducted in Spain with a sample size of 1743 students aged 12 to 20. The study revealed no discernible variation in nomophobia between adolescents and young adults [4]. Similarly, a cross-sectional study in Saudi Arabia indicated that younger people are more mobile phone-dependent than older people [12]. The difference in this study might be attributed to selection bias. Additionally, in this study, the result proved that participants with graduate degrees had lower mean nomophobia scores compared to those with high school or lower degrees. This is consistent with a study conducted in Turkey, which reported higher levels of nomophobia among high school students compared to graduates and working youth [28].

This result found that people from the Eastern region of Saudi Arabia had higher nomophobia scores compared to those from other regions. Additionally, people in the Eastern region also reported higher levels of psychological distress, which could be due to the sample size contrast, a different study found that the Northern region had the highest nomophobia scores among 41 participants compared to other regions [28].

Moreover, research conducted in Lebanon revealed elevated levels of severe nomophobia throughout all areas of the country [29].

This finding suggests that the size of the study sample may significantly influence our understanding of the prevalence of nomophobia and its related psychological distress in the community. A cross-sectional study conducted in Portugal with 495 young adults found a moderate positive correlation between nomophobia and depression, highlighting the link between the two (r=.374, p <.001) and anxiety (r =.340, p <.001) [30]. Another study involving 257 Croatian students aged 22 found that nomophobia was linked to a 12.4% increase in depression symptoms and a 14.5% rise in emotional stress among those exhibiting nomophobia symptoms [31]. Furthermore, a local cross-sectional study conducted with 335 participants in Saudi Arabia found a link between nomophobia and mild depression, with an average depression score of 8.55 (Z = 0.83) (SD = 3.41, ranging from 1 to 17) [24]. In addition, a local cross-sectional study examining nomophobia, anxiety, and related factors in the general populations of Saudi Arabia and Jordan found that 26.5% of participants experienced anxiety [11]. The study also revealed that the interquartile range (IQR) for phone usage was 210 minutes per day, with 51.2% of participants showing signs of dependency [11]. Overall, it can be concluded that cell phone dependence may incline nomophobia and subsequently correlate to psychological distress.

## Conclusion

This study on nomophobia and psychological distress in Saudi Arabia demonstrated a positive relationship between excessive mobile phone use and psychological distress, particularly among males, individuals with lower education levels, and residents of the Eastern region, who exhibited higher levels of both. The study had several strengths, including a relatively large sample size of 704 participants from various regions across Saudi Arabia, which enhanced statistical power and the likelihood that the results reflected the broader sample of the targeted population. Furthermore, this study is the first to investigate the relationship between nomophobia and psychological distress across multiple regions in Saudi Arabia, making a significant contribution to the existing body of knowledge. By examining this relationship in a culturally distinct setting, the research addresses an important gap in the literature and provides insights that are particularly relevant to the population residing in Saudi Arabia. The study’s findings not only add to the global understanding of how mobile phone dependency impacts mental health but also offer valuable context-specific evidence. This is especially crucial for understanding behavioral and psychological patterns in a rapidly developing nation like Saudi Arabia, where cultural norms, technological adoption, and social structures are unique. These insights pave the way for culturally tailored interventions and policy recommendations to address the psychological impacts of excessive mobile phone use within the country.

The study provides valuable insights but also presents several limitations. One significant limitation is the skewed sample composition, with 58.4% females and only 41.5% males, which may have limited the generalizability of the findings.

Another limitation is the use of snowball sampling, which could introduce selection bias. However, efforts were made to diversify the initial sample to mitigate the overrepresentation of specific subgroups, enhancing the generalizability of the results. While the cross-sectional design of the study inherently limits the ability to establish causal relationships between nomophobia and psychological distress, the inclusion of a large and diverse sample—704 participants from various regions of Saudi Arabia helped strengthen the findings by improving the robustness and generalizability of the results. Additionally, the use of multivariate analysis strengthened the study’s design by ensuring the statistical methods were appropriate. This approach ensured the reliability of the results by confirming that the data met the assumptions required for the analysis, thus reducing the risk of inaccurate conclusions and providing a stronger foundation for the study’s findings.

The study sheds light on the link between nomophobia and psychological distress in Saudi Arabia. However, it has methodological limitations, particularly in terms of sampling diversity, representativeness, and study design. Future research should focus on incorporating experimental designs, clinical interviews, and longitudinal approaches to better understand the long-term effects of nomophobia on psychological distress and provide stronger evidence for causal relationships.

Moreover, there is a noticeable gap in research focusing on children and preadolescents, despite the decreasing age at which individuals first own a mobile phone. Younger populations should be targeted more in future studies. Addressing this demographic could provide critical insights into early interventions and preventive strategies.

It is also crucial that nomophobia can be prevented through educational programs that instruct people in the appropriate use of technology. Currently, no programs specifically target nomophobia among adolescents and other vulnerable groups. It would be very beneficial if educational interventions could be developed in the future to reduce nomophobia behavior.

### Key points:

- Nomophobia is a condition in which a person becomes anxious because they fear lacking access to their mobile.
- This study found that nomophobia is common among adults in Saudi Arabia, with the majority of psychological distress symptoms.
- Sociodemographic factors such as being male and being Saudi were associated with higher nomophobia levels.
- Effective interventions to mitigate the widespread nomophobia and psychological distress are crucially needed.
- More studies are needed to better understand the impact of nomophobia on mental health.

## Supporting information

S1 Data Summary of the dataset variables. This dataset includes demographic variables, psychological assessment scores, and responses to survey questions related to smartphone use and mental health. Gender: Categorical variable indicating participant gender (Male, Female). Age: Age group of participants (coded numerically). Region: Region of residence (coded numerically). Nationality: Nationality of participants (coded numerically). Marital status: Marital status of participants (coded numerically). Educational status: Educational level of participants (coded numerically). Employment status: Employment status (coded numerically). Monthly income: Income category (coded numerically). Nomophobia score: Total score measuring dependency on smartphones. Psychological Distress Score: Total score assessing psychological distress. Survey Responses: Responses to psychological and behavioral questions related to smartphone use, stress, and mental health (measured on a Likert scale).
(XLSX)
